# COVID-19 and anxiety in pregnancy and postpartum: a longitudinal survey

**DOI:** 10.1186/s12889-025-22257-7

**Published:** 2025-03-26

**Authors:** Susan Ayers, Rose Meades, Andrea Sinesi, Helen Cheyne, Margaret Maxwell, Catherine Best, Stacey McNicol, Fiona Alderdice, Julie Jomeen, Judy Shakespeare, and the MAP Study Team, Georgina Constantinou, Simon Gilbody, Agnes Hann, Jennifer Holly, Grace Howard, Una Hutton, Rachael Leonard, Debra Salmon, Nazihah Uddin, James Walker, Louise R. Williams, Cassandra Yuill

**Affiliations:** 1 Centre for Maternal and Child Health Research, School of Health and Psychological Sciences, City, University of London, Northampton Square, London, EC1V 0HB UK; 2 Nursing, Midwifery and Allied Health Professions Research Unit, Pathfoot Building, University of Stirling, Stirling, FK9 4LA UK; 3 National Perinatal Epidemiology Unit, Oxford Population Health, Old Road Campus, Headington, Oxford, OX3 7LF UK; 4 Southern Cross University, Gold Coast Airport, Terminal Drive, Bilinga, QLD 4225 Australia; 5 Retired General Practitioner, Oxford, OX2 7AG UK

**Keywords:** COVID-19, Pandemic, Anxiety, Pregnancy, Postpartum

## Abstract

**Background:**

Anxiety is estimated to affect between 15 and 20 per cent of women during pregnancy and postpartum. The COVID-19 pandemic resulted in wide-ranging changes to how people lived, worked and socialised around the world. COVID and pandemic-related restrictions to maternity services may have exacerbated anxiety during pregnancy and the postnatal period. This study aimed to determine: (1) levels of COVID exposure and perceived risk; (2) adherence to Government guidelines and restrictions; and (3) the impact of COVID and COVID-related restrictions on perinatal anxiety and mental health in the UK.

**Methods:**

A longitudinal survey (*n* = 2122) of COVID and anxiety in women during early pregnancy, mid-pregnancy, late pregnancy and postpartum.

**Results:**

38.41% of participants had COVID before or during the study. Perinatal anxiety was predicted by participants having poor general health, being of Asian or mixed ethnicity, having previous mental health problems, believing that COVID would make them severely ill, and reporting that COVID had impacted on their mental health. Over time, more women were infected with COVID, and the perceived severity of COVID decreased. Experiencing mild COVID was associated with decreased anxiety at the subsequent time point (mean difference -0.72, 95% CI -1.38 to -0.07, *p* = 0.030). Very few participants in this sample had severe COVID (2.9%) or reported it having a severe impact on their mental health (5.66%). Most participants (75.3%) said the pandemic had ‘no’ or a ‘slight’ impact on their mental health. Pandemic-related restrictions to maternity care affected more women, with around 40% reporting anxiety about being separated from baby, their partner not being with them in labor, or having to leave shortly after the birth. Level of adherence to guidelines was variable, depending on the restriction.

**Conclusions:**

Findings suggest pandemic-related restrictions caused anxiety for more women than COVID per se. Adherence to guidelines was variable yet the prevalence of COVID infections was low compared to the general population. Findings can be used to inform policy and practice for future pandemics and health-related crises.

**Supplementary Information:**

The online version contains supplementary material available at 10.1186/s12889-025-22257-7.

## Background

TheCOVID-19 pandemic affected all aspects of peoples’ lives during its peak period from 2020 to 2022. Restrictions imposed by governments limited social contact and movement. Pressures on healthcare services and rapid changes to guidance, policy and protocols during this time not only impacted on the day-to-day running of maternity services but also on women and families who used these services. Qualitative research suggests a wide-ranging impact of pandemic-related changes on pregnant and postnatal women and their families [1]. A review of the qualitative literature showed pregnant women reported anxiety and fear, being isolated and social support being disrupted, feeling as if they were ‘going it alone’, and anticipatory grief and despair about giving birth in a crisis. Postpartum, women reported feeling isolated, facing disruptions to postnatal care, and that the experience of COVID and related traumas would affect them for years to come [1]. However, this review also identified positive benefits to the social restrictions at this time, such as the father of the baby being at home more [1].

Unsurprisingly, there is substantial evidence the pandemic and associated changes had an effect on women’s mental health during pregnancy and postpartum [2–10]. For example, a comparison of population-based surveys in the UK found self-reported postnatal depression prevalence increased from 16% pre-pandemic to 24% during the pandemic [11]. Similarly, a study of over 14,000 women in Italy found an increase in postnatal depression from 11.6 to 25.5% [12]. Multiple reviews and meta-analyses support an increase in postnatal depression, with pooled prevalence ranging from 17 to 24 percent [2–10].

The impact of the pandemic on anxiety is less clear, with research showing mixed results. Some studies suggest anxiety was higher than depression, affecting around 40% of women [8]. For example, a study of over 3,000 pregnant and postpartum women in Spain found 47% reported perinatal anxiety [13]. In contrast, other studies found only small increases in anxiety compared to pre-pandemic levels [14]. Reviews of this evidence are also mixed, concluding that the pandemic increased anxiety [4, 5, 8–10], did not increase anxiety [15], or that the evidence is inconsistent [16]. The latter review [16] focused solely on perinatal anxiety and concluded the literature is equally split, with around half of studies (8 out of 15) reviewed showing an increase. This review also highlighted the comparatively few studies on perinatal anxiety and restriction of some studies to specific populations (e.g. minority groups) [17] or convenience samples [18].

The inconsistent research on perinatal anxiety during the pandemic is likely due to the limited research, heterogeneity in methodology (e.g. different methods of sampling or assessment), contextual issues such as timing during the pandemic, and cross-cultural variation in factors such as severity and restrictions in place. For example, there is some suggestion that the prevalence of antenatal anxiety during the pandemic was higher in Europe compared to Asia [8, 19]. There is also limited information on the extent to which women adhered to Government-mandated or advised restrictions during pregnancy and postpartum, and how this influenced their mental health. This information is important in order to determine the impact of restrictions on physical and mental health, as well as to inform future pandemic-type situations. There are some indications that adherence to Government restrictions was associated with poorer mental health [20]. It is also possible that some increased anxiety is a normal reaction to a pandemic, which is not necessarily problematic and may be temporary.

The Methods of Assessing Perinatal Anxiety (MAP) study [21] was a prospective, longitudinal study of anxiety during pregnancy and postpartum conducted in the U.K. from 2020 to 2022. After the start of the pandemic measures of COVID exposure, perceived risk and adherence to U.K. Government restrictions were added to the MAP study prior to data being collected. The analyses reported in this paper aimed to determine levels of COVID exposure and perceived risk, the impact of COVID, COVID-related Government restrictions, and adherence to Government restrictions on perinatal anxiety and mental health.

## Methods

### Design

A longitudinal survey conducted between November 2020 and October 2022 in England and Scotland. Participants completed questionnaire measures in early pregnancy (mean 11.4 weeks, SD 2.0), mid-pregnancy (mean 23.0 weeks, SD 1.3), late pregnancy (mean 31.9 weeks, SD 1.2) and postpartum (mean 7.9 weeks, SD 2.4). The study was pre-registered [21] and the protocol available online [22].

### Sample

Participants were recruited between November 2020 and October 2021 through 12 National Health Service (NHS) Trusts in England and five NHS Health Boards in Scotland. Women were eligible for the MAP cohort if they were: aged 16 years or over; less than 15 weeks pregnant at the time of recruitment; able to provide written informed consent; and had sufficient English to understand and complete questionnaires. A total of 2243 women took part and 2199 responded in early pregnancy (98%), 1495 responded in mid-pregnancy (67%), 1400 responded in late pregnancy (62%) and 1368 responded postpartum (61%). Completion of the COVID measures was optional and 2,122 participants completed the COVID questionnaire for at least one timepoint, with a total of 5,856 COVID questionnaires completed over all four timepoints combined.

### Measures

The MAP survey included measures of anxiety, distress, general health, quality of life, social support and sociodemographic characteristics [21]. In addition, questions were asked about COVID exposure, the impact of the COVID pandemic on mental health, and participants’ adherence to Government guidance and restrictions. Measures analysed and reported in this paper are detailed below:

#### COVID exposure and perceived risk

For exposure to COVID, participants were asked to indicate if they, their baby, family, friends or colleagues had had COVID and whether this was mild, moderate or severe (irrespective of whether they received a test). Participants were asked about perceived risk of COVID with the question ‘How likely do you think it is that either you, your baby or someone close to you will become infected with COVID-19?’ and the perceived severity of this risk by ‘How likely do you think it is that either you, your baby, or someone close to you will be severely ill if infected with COVID-19?’ For both these questions the response options were Very likely/Likely/Uncertain/Unlikely/Very unlikely.

#### Impact of the pandemic on mental health

The impact of the pandemic on mental health was assessed by asking participants ‘How much has the COVID-19 pandemic impacted your mental health e.g. depression, anxiety, or any other mental health problem?’ with response options of: No impact/Slight impact/Moderate impact/severe impact. Participants were then asked to rate how specific pandemic-related changes and restrictions impacted on their mental health. Changes asked about are shown in Table 1. Instructions were ‘Please tell us whether the following COVID-19 related changes have made you feel anxious’ and response options were: Not at all anxious/Slightly anxious/Moderately anxious/Very anxious/Extremely anxious or Not applicable. Questions were based on expert consensus, public and patient involvement and information on relevant websites e.g. Royal College of Midwives, Royal College of Obstetricians and Gynaecologists.

**Table 1:** Pandemic-related changes and restrictions assessed

Maternity care changes or restrictions
Disrupted antenatal/postnatal appointments
Lack of information or inconsistent information from healthcare professionals
Lack of antenatal classes
Attending hospital for antenatal or postnatal care
Changes to where they can give birth (e.g. not being able to go to a birth centre)
Being in hospital to have the baby
Concerns about not having their partner/birth partner with them during birth
Concerns about their partner having to leave the hospital soon after the birth
Concerns about being separated from their baby
Concerns about breastfeeding
Availability of formula milk/baby supplies
**Personal changes**
Isolation
Not having support from family and friends
Impact on family finances
Changes to mental health appointments
**COVID-related concerns or changes**
Concerns about themselves or others close to them being infected with COVID
Uncertainty about the impact of the virus

#### Adherence to Government guidance

Participants were asked about adhering to Government guidance in place in 2020. Participants were asked ‘To what extent have you, or did you, adhere to Government guidance on:’ and provided with a list of 10 rules from UK guidelines in place in 2020. These were:

- Personal hygiene (2 items): (1) Handwashing; (2) Using a tissue when anyone in your house sneezes or coughs, discarding it and washing your hands.
- Physical distancing (8 items): (1) Physical distancing; (2) Avoiding any gatherings with friends and family; (3) Keeping in touch with friends and family using phone, internet and social media rather than face to face; (4) Avoiding large and small gatherings in public spaces; (5) Working from home where possible; (6) Self-isolating if you or someone you live with has symptoms; (7) Avoiding nonessential use of public transport; (8) Using telephone or online services to contact your GP or other essential services.

Response options for these items were: not at all; slightly; moderately; mostly; completely; or not applicable.

Anxiety was measured using the Stirling Antenatal Anxiety Scale (SAAS) [23], a 10-item, clinically derived questionnaire developed specifically to assess perinatal anxiety. The SAAS includes general anxiety and pregnancy-specific anxiety items. The pregnancy-specific items are about the birth and baby so the scale can be used postnatally. Items are scored on a 0–4 Likert scale with a range of 0–40 [23]. The scale has good diagnostic accuracy and performs well at identifying women with perinatal anxiety [24].

Sociodemographic characteristics were measured using standard classifications for England or Scotland [25, 26]. Characteristics measured included age, ethnicity, education and relationship status.

Medical history and obstetric information was measured by self-report and included previous history of mental health problems (defined as a response of yes to the question ‘have you ever experienced psychological or mental health problems?’) and preterm birth (defined as a response of yes to the question ‘was your baby (or babies) born prematurely?’).

General health was measured using the visual analogue scale of the EuroQoL EQ-5D-5L [27] completed in late pregnancy. The EQ5D is a preference-based measure of health status and quality of life widely used in health research [28].

### Procedure

Participants were recruited in 12 NHS Trusts in England and five NHS Health Boards in Scotland. Recruitment was conducted in person or remotely by research or clinical midwives/nurses, usually around the time of a woman’s pregnancy booking appointment or early pregnancy scan. If women were interested in taking part they could give informed consent at that point and be given the first questionnaire to complete, or give consent for their contact details to be shared with the research team who then contacted them directly to provide further information, answer questions, and obtain informed consent. Questionnaires were sent to participants at three timepoints in pregnancy and once postpartum. NHS sites were contacted before sending questionnaires to check if any serious adverse events had occurred (e.g. pregnancy loss, stillbirth). Questionnaires were completed online or by post, depending on participants’ preferences. Safeguarding procedures were in place for any participant who scored above the cut-off on the NICE-recommended measure for perinatal anxiety and/or if they expressed suicidal intent. Participants who did not return their questionnaires were followed up by email, letter or telephone up to a maximum of three times.

### Analysis

Frequencies for demographic variables were presented as number and percentage in each category. For other outcomes descriptive numbers and percentages are given for each time point. Analysis of relationship between perinatal stage (early, mid, late or postnatal) and the odds of ever having COVID was conducted using mixed effects logistic regression model with ‘never’ versus ‘ever’ having COVID analysed as a binary dependent variable, timepoint as an independent factor variable, and the random intercept included to account for repeated measures on individuals. Mixed effects models are a common method for analysing longitudinal or panel data and use data from all time points in a single analysis.

Difference between perinatal stages in the perceived severity of illness from COVID infection was examined using a mixed effects ordinal regression model.

Relationships between experiencing COVID infection, perceived likelihood of infection, perceived severity of likely infection, and anxiety at the same perinatal stage were assessed using a series of mixed effects linear regression models with total anxiety scores (SAAS) as the continuous dependent variable. COVID-related independent variables were included as factor variables, and a random intercept for individuals was included in the model to account for repeated measures. In addition, to investigate the relationship between COVID-related independent variables and subsequent anxiety, lagged variables were created and included as the independent variables. That is, the value of the independent variable (e.g. perceived severity of COVID infection) at the previous time point was used to predict the dependent variable of anxiety (SAAS score) at the subsequent timepoint, leveraging the longitudinal nature of the dataset. To further investigate the effect of change in COVID status we created a variable that represented a change from no COVID at the previous time point to mild COVID at the current time point.

A fully adjusted mixed effects model was used to examine the mutually adjusted impact of COVID infection, perceived likely severity of COVID infection and perceived risk of infection adjusted for age, ethnicity, previous mental health problems (reported in early pregnancy), and general health (reported in late pregnancy) on anxiety symptoms.

In order to investigate the relationship between adherence to COVID behavioral guidance and the likelihood of experiencing a COVID infection by the next timepoint we again used mixed effects models with COVID infection as the binary outcome and a lagged variable representing reported adherence to guidelines at the previous timepoint as the independent variable. These analyses were adjusted for age, relationship status, ethnicity, previous mental health problems (reported in early pregnancy). In order to explore the effects of the stage of the pandemic on outcomes, a variable was created which divided the recruitment period into 4-month intervals. All analyses were adjusted for this variable and the results are shown in supplementary materials. Odds ratios and proportions are reported with 95% confidence intervals (CI) where appropriate.

Analyses were conducted in Stata version 16 for Windows or higher.

## Results

Characteristics of participants who completed the COVID questionnaire are given in Table 2. Mean age of participants was 31.5 years (SD 5.1, range 16 to 50), which is similar to the standardised mean age for women giving birth in England and Wales (30.9 years) at that time [29]. The sample was more ethnically diverse and highly educated than the UK population [25, 26], with a greater proportion of our sample from non-White ethnic groups (22.65%) and having university education (61.63%).

**Table 2:** Sample characteristics

		Freq	Percent
Highest education	School or A-levels or equivalent	765	38.21
Level	University undergraduate degree	817	40.81
	University postgraduate degree	420	20.98
	Total	2002	100.00
Ethnic group	White	1545	77.17
	Mixed/multiple ethnic groups	91	4.55
	Asian/Asian British	258	12.89
	Black/African/Caribbean/Black British	88	4.40
	Other ethnic group	20	1.00
	Total	2002	100.00
Number of children	0	1055	51.41
	1	713	34.75
	2	190	9.26
	3	68	3.31
	4 +	26	1.27
	Total	2052	100.00
Relationship status	In a relationship, non-cohabitating	162	8.15
	Cohabitating	680	34.21
	Married/Civil partnership	1067	53.67
	Separated/Divorced/Single	79	3.97
	Total	1988	100.00

### COVID exposure and perceived risk of COVID

Participants’ exposure to COVID over time is shown in Table 3. Unsurprisingly, the proportion of participants who reported ever having had COVID increased across timepoints. The odds of having had COVID at the postnatal timepoint relative to the early pregnancy timepoint was 7.64 (95% CI 5.40 to 10.82) with 1 in 5 participants (21.51%, 95% CI 19.51 to 23.62%) reporting ever having had COVID in early pregnancy and over 1 in 3 participants.

**Table 3:** Exposure to COVID and perceived risk of COVID at different timepoints

		Early pregnancy		Mid-pregnancy		Late pregnancy		Postpartum	
		Freq	Percent	Freq	Percent	Freq	Percent	Freq	Percent
Participants who had experienced a COVID infection	No COVID	1244	78.49	838	76.18	639	69.08	441	61.59
	Mild	181	11.42	142	12.91	171	18.49	179	25.00
	Moderate	136	8.58	109	9.91	107	11.57	81	11.31
	Severe	24	1.51	11	1.00	8	0.86	15	2.09
	Total	1585	100.00	1100	100.00	925	100.00	716	100.00
Perceived risk of them, their baby or someone close to them getting COVID	Very unlikely	273	13.55	129	9.41	102	8.15	110	9.47
	Unlikely	401	19.90	181	13.20	172	13.74	144	12.40
	Uncertain	887	44.02	583	42.52	519	41.45	388	33.42
	Likely	353	17.52	326	23.78	307	24.52	306	26.36
	Very likely	101	5.01	152	11.09	152	12.14	213	18.35
	Total	2015	100.00	1371	100.00	1252	100.00	1161	100.00
Perceived risk of them, their baby or someone close to them being severely ill with COVID	Very unlikely	349	17.36	205	14.94	161	12.90	207	17.86
	Unlikely	611	30.40	344	25.07	395	31.65	387	33.39
	Uncertain	843	41.94	671	48.91	568	45.51	458	39.52
	Likely	161	8.01	124	9.04	96	7.69	78	6.73
	Very likely	46	2.29	28	2.04	28	2.24	29	2.50
	Total	2010	100.00	1372	100.00	1248	100.00	1159	100.00

(38.41%, 95% CI 34.83 to 42.08%) reporting ever having had COVID postpartum. Very few participants reported having severe COVID: ranging from 1.51% in early pregnancy to 2.09% postpartum.

Perceived risk of being infected by COVID increased over time, with 22.53% stating it was ‘likely’ or ‘very likely’ they would be infected in early pregnancy, increasing to 44.71% postnatally. A mixed effects ordinal logistic regression model indicated the odds for perceiving they were more likely to become infected at the postnatal timepoint compared to early pregnancy was OR 3.05 (95% CI 2.63 to 3.55). However, the perceived risk of being severely ill with COVID dropped slightly over time, from 10.3% stating ‘likely’ or ‘very likely’ in early pregnancy to 9.23% postnatally, possibly reflecting the availability of vaccines, greater attenuation of the virus type and/or greater public knowledge. A mixed effects ordinal regression model indicated the odds of perceiving greater risk at the postnatal time point compared to early pregnancy was OR 0.82 (95% CI 0.70–0.95).

### Impact of COVID

Experiencing mild COVID was associated with reduced anxiety. Compared to participants who never had COVID, those who had mild COVID had significantly lower anxiety symptoms at the subsequent timepoint (mean difference −0.72, 95% CI −1.38 to −0.07, *p* = 0.030). No significant differences were found for moderate or severe COVID (mean difference −0.55, 95% CI −1.29 to 0.20; and 0.65, 95% CI −1.22 to 2.51 respectively). The effect of mild COVID on anxiety at the same timepoint showed the same pattern and these effects remained significant after adjusting for relationship status, general health, ethnicity, age, and previous mental health problems (Table 4). Change in infection status between time points (i.e. the transition from an individual having no previous COVID infection to having had COVID in a mild form) was associated with a small decrease in anxiety at that time point, which was not statistically significant (−0.12 95% CI −0.84 to 0.60).

**Table 4:** Perceived severity of COVID and anxiety at the same timepoint

		Anxiety symptoms		
	N	Mean (SD)	Coefficient	Adjusted coefficient
No COVID	3143	9.23 (7.29)	Ref	ref
Mild COVID	671	8.32 (6.98)	−0.66 (−1.19 to −0.13)*	−0.58 (−1.10 to −0.05)*
Moderate COVID	433	9.68 (7.52)	−0.08 (−0.71 to 0.55)	−0.44 (−1.06 0.18)
Severe COVID	37	10.71 (8.45)	0.07 (−1.50 to 1.64)	−0.31 (−1.84 to 1.23)

^a^Adjusted for age, marital status, ethnic group, general health and previous mental health problems

*p < 0.05

Women who thought it was ‘likely’ or ‘very likely’ that they, their baby or someone close to them would become infected with COVID had greater anxiety at the following timepoint compared to women who thought it unlikely (mean difference 0.76, 95% CI 0.01 to 1.52; mean difference 1.13, 95% CI 0.24 to 2.02 respectively). Women who thought they, their baby or someone close to them would be severely ill if infected with COVID also had greater anxiety at the following timepoint than those who reported they were unlikely to be severely ill (mean difference 2.23, 95% CI 0.86 to 3.60).

The perceived impact of COVID on mental health across timepoints is shown in Table 5. This shows that most participants reported ‘no’ or a ‘slight impact’ of the pandemic on their mental health (71.94%, 71.68%, 72.86% and 75.3% in early, mid-, late pregnancy and postpartum respectively). A moderate or severe impact of COVID on mental health was reported by just over 1 in 4 women (e.g. 28.06% in early pregnancy). Very few women reported a severe impact (5.27–5.66%). These proportions remained stable through pregnancy and postpartum. A regression model indicated that, within individuals, the odds of reporting an impact of COVID on mental health declined over timepoints (OR 0.90, 95% CI 0.84 to 0.94).

**Table 5:** Perceived impact of the pandemic on mental health

	Early pregnancy		Mid-pregnancy		Late pregnancy		Postpartum	
	Freq	%	Freq	%	Freq	%	Freq	%
No impact	473	23.50	266	19.42	268	21.53	304	26.25
Slight impact	975	48.44	716	52.26	639	51.33	568	49.05
Moderate impact	451	22.40	312	22.77	272	21.85	225	19.43
Severe impact	114	5.66	76	5.55	66	5.30	61	5.27
Total	2013	100.00	1370	100.00	1245	100.00	1158	100.00

An adjusted mixed effects model was conducted to determine which COVID-related variables were most predictive of anxiety in a mutually adjusted model (Table 6). Perinatal anxiety was most predicted by participants believing COVID would make them severely ill, and reporting that COVID had impacted on their mental health. Exposure to COVID and perceived risk of catching COVID was not significantly associated with anxiety in the multivariate model. Adjusted variables that were significantly associated with anxiety were participants from Asian/British Asian or mixed ethnicity backgrounds, poor general health and a history of mental health problems.

**Table 6:** Adjusted mixed effects model of COVID exposure, perceived risk, and impact on anxiety symptoms

Anxiety (SAAS total score)	Coef	Standard Error	t-value	*p*-value	95% Confidence Interval	Sig	
**Time point**							
Early pregnancy	0						
Mid pregnancy	−1.461	.189	−7.73	< 0.001	−1.831	−1.09	***
Late pregnancy	−2.111	.203	−10.40	< 0.001	−2.509	−1.713	***
Postpartum	−1.693	.231	−7.34	< 0.001	−2.145	−1.241	***
**Ethnic group**							
White	0						
Mixed/multiple ethnic background	1.987	.678	2.93	.003	.658	3.316	***
Asian/Asian British	1.305	.44	2.97	.003	.443	2.166	***
Black/African/Caribbean	.608	.763	0.80	.426	-.888	2.103	
Other ethnic group	-.079	1.57	−0.05	.96	−3.155	2.998	
**Age**	-.075	.026	−2.84	.004	-.127	-.023	***
**Previous mental health problems**	3.53	.278	12.69	< 0.001	2.985	4.075	***
**COVID exposure**							
No COVID	0						
Mild COVID	.044	.265	0.17	.869	-.476	.564	
Moderate COVID	-.092	.309	−0.30	.765	-.698	.513	
Severe COVID	-.908	.759	−1.20	.232	−2.396	.58	
** General health**	-.1	.006	−17.16	< 0.001	-.112	-.089	***
**Perceived risk of them, their baby or someone close to them getting COVID**							
Unlikely	-.413	.415	−1.00	.32	−1.226	.4	
Uncertain	-.153	.403	−0.38	.705	-.943	.638	
Likely	-.222	.42	−0.53	.597	−1.046	.601	
Very likely	.151	.462	0.33	.743	-.753	1.056	
**Perceived risk of them, their baby or someone close to them being severely ill with COVID**							
Unlikely	.161	.318	0.51	.612	-.462	.784	
Uncertain	.616	.334	1.84	.066	-.04	1.271	*
Likely	1.436	.433	3.31	.001	.587	2.286	***
Very likely	1.177	.643	1.83	.067	-.084	2.438	*
**Impact of COVID on their mental health**							
Slight impact	1.481	.254	5.82	0	.983	1.98	***
Moderate impact	3.6	.314	11.46	0	2.984	4.215	***
Severe impact	5.811	.467	12.44	0	4.895	6.726	***
Mean dependent variable	8.967			SD dependent var		7.196	
Number of observations	3806			Chi-square		1257.089	
Prob > chi2	0.000			Akaike crit. (AIC)		23,574.619	

Adjusted for relationship status, ethnicity, age, general health and previous mental health problems Information for adjusted variables that were significant is included in the table

^***^p < .01

^**^p < .05

^*^p < .1

### Impact of COVID-related restrictions

**Fig. 1:**
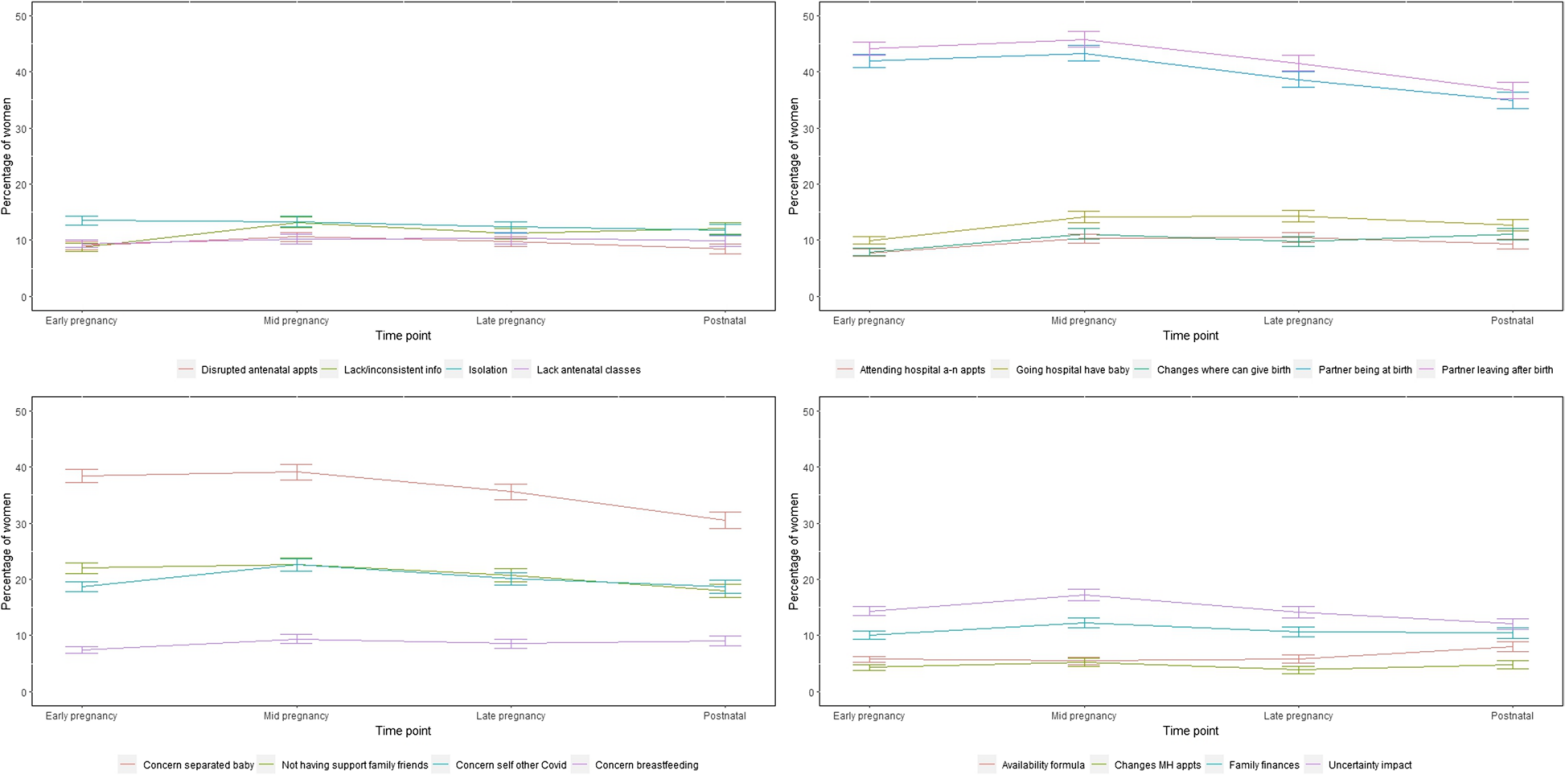
Proportion of sample who were very or extremely anxious about COVID-related restrictions

Restrictions to antenatal and postnatal care during the pandemic affected women’s experiences and caused anxiety. Figure 1 shows the proportion of participants who reported the restrictions made them very anxious or extremely anxious with 95% confidence intervals for the proportions at each time point. The restrictions that caused anxiety to most participants were: partners not being present during labour; partners having to leave shortly after the birth; and being separated from the baby. These were reported by around 40% of participants as making them very or extremely anxious. Around 20% of participants were very or extremely anxious about the risk of COVID for themselves and/or others, and not having the support of family and friends. Fewest participants were anxious about the availability of infant formula or changes to mental health appointments, which were reported by around 5% of participants as making them very or extremely anxious.

### Adherence to Government guidance on COVID

**Fig. 2:**
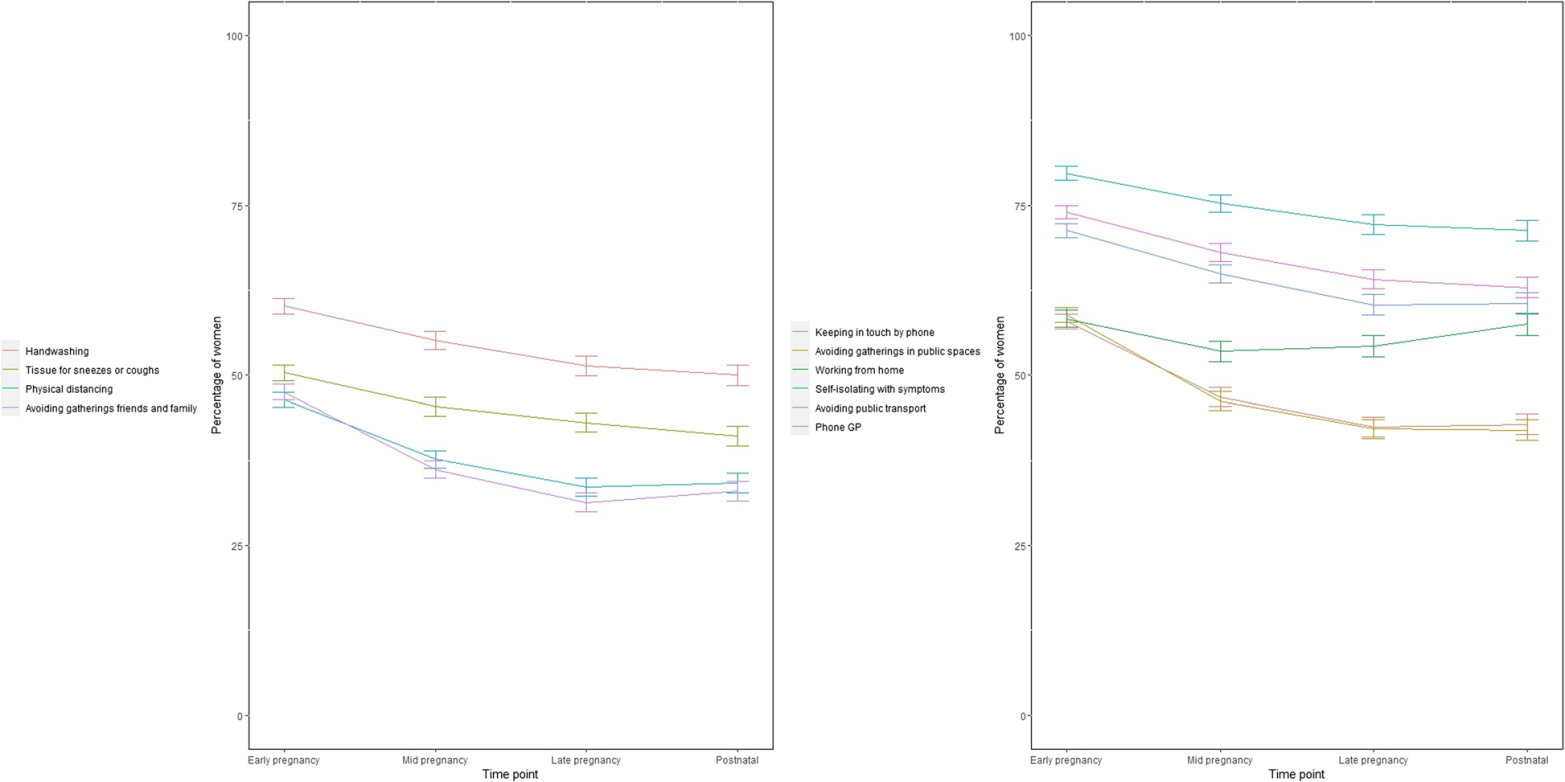
Proportion of participants adhering to Government guidance and restrictions

The extent of adherence to Government guidance and restrictions is shown in Fig. 2 with 95% confidence intervals for the proportions at each time point. This shows that 40 to 80% of participants adhered to Government guidelines, depending on the guideline. There was some decrease in adherence over time. The guidelines fewest participants (around 40%) adhered to were avoiding public gatherings, avoiding gatherings with friends and family, physical distancing and keeping in touch by phone or other remote methods.

Level of adherence to guidelines was not associated with levels of anxiety in participants, with one exception. Those who completely followed guidance to avoid meeting or gathering with friends and family reported significantly more anxiety compared to those who did not follow this guidance (mean difference 1.02, 95% CI 0.21 to 1.83).

Non-adherence to guidance was associated with greater likelihood that participants reported having had COVID at the next questionnaire timepoint. Greater likelihood of having had COVID was observed in participants who reported not avoiding public transport (aOR 5.15, 95% CI 1.62 to 16.34), not working from home (aOR 3.24, 95% CI 1.69 to 6.21) when comparing those who adhered to these restrictions completely versus not at all. Greater likelihood of having had COVID at the next timepoint was observed in those not using phone or other remote methods to keep in touch with friends and family (aOR 2.58, 95%CI 1.75 to 3.81), not avoiding social gatherings with family and friends (aOR 1.86, 95% CI 1.25 to 2.76) or not avoiding public gatherings (aOR 1.80, 95% CI 1.24 to 2.62) when comparing those who adhered to these mostly compared to completely. ‘Mostly’ is used as the comparison for the latter outcomes because of the low frequency of respondents reporting not adhering to these guidelines at all.

Adjusting for the stage of the pandemic when participants were recruited did not lead to any substantive difference in results (see Supplementary material).

## Discussion

This was a large, longitudinal survey which examined COVID exposure and perceived risk, the impact of COVID, COVID-related Government restrictions, adherence to Government restrictions and perinatal anxiety. Findings confirm that over the course of the survey increasing numbers of participants were infected with COVID and the perceived severity of COVID and perceived impact on mental health decreased. Perinatal anxiety was predicted by participants having poor general health, being of Asian or mixed ethnicity, having experienced previous mental health problems, believing that COVID would make them severely ill, and reporting that COVID had impacted on their mental health. The more severe women perceived COVID to be, the more anxiety they reported. Generally, this anxiety decreased after women had been infected with COVID. Pandemic-related restrictions to maternity care affected a greater proportion of participants’ than COVID per se, with around 40% reporting feeling very or extremely anxious about their partner not being with them in labour, their partner having to leave shortly after the birth, or being separated from baby, compared to 5.66% reporting COVID had a severe impact on their mental health.

Adherence to government restrictions was not absolute and the level of adherence was mixed, depending on the restriction. Unsurprisingly, non-adherence was associated with greater likelihood of having COVID. However, adhering to guidelines to avoid friends and family was associated with increased anxiety. This impact of pandemic-related restrictions on anxiety is consistent with a UK study of over 1,700 people that found the more strictly people complied with COVID restrictions during the pandemic the worse their mental health was post-pandemic [20]. Similarly, women’s anxiety in this study caused by worries about their partner not being with them during labour or after the birth, or being separated from their baby is consistent with themes identified by reviews of qualitative research [1, 30] of pregnant and postnatal women feeling as if they were ‘going it alone’, ‘abandoned’, and feeling despair about giving birth in a crisis.

Very few participants in this sample had severe COVID (2.9%) or reported it as having a severe impact on their mental health (5.66%). Most participants said the pandemic had no or only a slight impact on their mental health and this finding was stable across time suggesting it was robust. This is in contrast to qualitative and quantitative research suggesting the pandemic had a large impact on perinatal mental health, particularly depression [2–10], and contributes to inconsistent findings of the impact of the pandemic on anxiety [15]. This inconsistency may be due to methodological issues such as previous studies being cross-sectional, use of different measures, or sampling biases. It is also compounded by the rapid generation of knowledge from a wide range of countries and contexts. The inconsistency is reflected in reviews of the impact of the pandemic on perinatal mental health which conclude either that the pandemic increased anxiety [4, 5, 8–10], did not increase anxiety [15], or that the evidence is inconsistent [16]. The findings of this current study suggest these inconsistencies may be because the relationship between COVID and perinatal anxiety is mediated by pandemic-related restrictions, rather than COVID per se.

However, the low impact of COVID on mental health may be because the prevalence of COVID infections (38.4%) in this sample was less than the prevalence in the general population at the same time (70.7% England, 51.5% Scotland) [31]. It is not clear why this was the case. It is possible that being pregnant and having a very young baby meant women were more cautious and motivated to avoid catching COVID. Although findings suggest over half of participants did not adhere to some restrictions, particularly those of avoiding friends, family or social gatherings, adherence may be higher in this group than in the general population. Another explanation is that maternity leave might be protective because some women would not have gone into the workplace who might have done, despite working from home being widespread. The finding that women from Asian and mixed ethnic backgrounds reported greater perinatal anxiety suggests specific minority groups were more affected. This is consistent with evidence showing those from Asian and Black ethnic minority groups are at greater risk of maternal morbidity and mortality [32, 33], and poorer outcomes following COVID infection [34].

### Strengths and limitations

The strength of this study was that it was a large, population-based survey with measures completed at multiple timepoints in pregnancy and postpartum. It therefore provides a broadly representative view of the impact of the COVID pandemic on pregnant women during this time. It was also longitudinal so offers insight into changes in COVID, perceived risk and impact over the course of pregnancy and postpartum, including women’s behaviour in adhering to pandemic restrictions. In doing so it highlights which aspects of maternity care and daily life are important to women during pregnancy and postpartum.

Various limitations need to be considered. The sample was more highly educated than the general population so results may not be generalisable. Measurement was via self-report and the inclusion of COVID questions in the MAP study was opportunistic, based on the pandemic occurring as the MAP study started, and the measures had to be developed quickly. The impact of the pandemic on mental health was therefore measured with one item asking participants to report the extent to which the pandemic had impacted on their mental health overall. The advantages and disadvantages of single-item measures have been widely debated [35] but results are not directly comparable to research using different measures. Similarly, the measures of the impact of COVID on mental health and anxiety due to COVID-related restrictions were not equivalent, making direct comparisons difficult. The course of the pandemic and Government restrictions varied during the period of data collection. Whilst we were able to adjust for the stage of the pandemic, this was done in 4-month blocks, and we were not able to adjust for changes in Government restrictions. The nuances of anxiety and behaviour during the pandemic are therefore unlikely to have been captured.

Such difficulties are similar to other COVID research conducted at this stage in the pandemic when validated measures were not available. The government restrictions were specific to the UK at the time the survey was conducted. However. the focus on personal hygiene and physical distancing is similar to restrictions implemented in other countries. Finally, the pandemic and restrictions evolved and changed over time, as did the availability of vaccines, all of which would have influenced women’s emotional and behavioral responses and adherence to guidelines. It should be noted that although there were statistically significant relationship between, for example, COVID infection and anxiety, the absolute magnitude of these relationships was small and may not be clinically significant.

### Implications for practice and policy

Knowledge about the impact of pandemics and associated restrictions is important for future policy and practice during pandemics and other crises that affect healthcare services. This study suggests pandemic-related restrictions caused anxiety for many more women than COVID itself. However, it has to be recognised that these restrictions played an important part in reducing the number of infections and deaths. Based on the results of this and other studies [30], we recommend that restrictions that caused high levels of anxiety (i.e. preventing partners’ attendance at labour/birth and being separated from the baby) should be carefully considered in future pandemics or similar healthcare crises and implemented only if absolutely necessary. The nature and impact of these restrictions needs to be considered alongside the associated costs for mental health in pregnancy and longer term.

Another implication is that targeted information and support may be helpful for those more vulnerable to perinatal anxiety under these circumstances. The current study suggests vulnerability factors in relation to anxiety are previous mental health problems, poor health and Asian or mixed ethnicity.

## Conclusion

This study was one of a few population-based longitudinal studies examining the impact of the COVID-19 pandemic on perinatal anxiety. Perinatal anxiety was greatest in participants of Asian or mixed ethnicity, reporting poor general health, previous mental health problems, who believed COVID would make them severely ill, and reported that COVID had impacted on their mental health. Findings suggest pandemic-related restrictions caused more participants to be anxious than COVID per se, and that as more women were infected with COVID, perceived severity and associated anxiety decreased. Adherence to guidelines was not absolute and the level of adherence to guidelines was mixed. However, very few participants in this sample had severe COVID (2.9%) or reported it as having a severe impact on their mental health (5.66%). The majority of women in this sample said the COVID pandemic had no or a slight impact on their mental health. Findings can be used to inform policy and practice for future pandemics and health-related crises.

## Supplementary Information

Supplementary Material 1.

## Data Availability

The datasets used and analysed during the current study are available from the corresponding author on reasonable request. The study protocol is available here https://njl-admin.nihr.ac.uk/document/download/2034506.

## Abbreviations

CI: Confidence Interval
COVID-19: Coronavirus Disease 2019
EQ5D: EuroQoL measure of quality of life
GAD: Generalised Anxiety Scale
MAP: Methods of Assessing Perinatal anxiety study
NHS: National Health Service
OR: Odds Ratio (aOR adjusted Odds Ratio)
SAAS: Stirling Antenatal Anxiety Scale
SD: Standard Deviation
UK: United Kingdom
