# Supplementary material for: COVID-19 and anxiety in pregnancy and postpartum: a longitudinal survey
Source: BMC Public Health. 2025 Mar 26;25:1146. doi: 10.1186/s12889-025-22257-7 (PMC11938643; doi:10.1186/s12889-025-22257-7)
Supplement: Supplementary file 1 — Supplementary Material 1. [file 12889_2025_22257_MOESM1_ESM.docx]

# Supplementary material

This supplementary file shows the effect of adjusting the reported analyses for the recruitment time period to account for variation in the COVID pandemic during the course of the survey. Recruitment time was broken down into four periods each of approximately 4 months duration and these were included in the analyses as a dummy coded factor variable.

Table S1 shows the effect of adjusting the reported mixed effects analyses for the recruitment time period.

**Table S1. mixed effects analyses adjusted for the recruitment time period**

| **Analysis** | **Original effect** | **With adjustment for recruitment window** |
| --- | --- | --- |
| Any COVID by perinatal timepoint | 7.64 (5.40-10.82) | 7.88 (5.55 to 11.20) |
| Likelihood participant was infected by the perinatal timepoint | 3.05 (95% CI 2.63 to 3.55). | 3.02 (2.59 to 3.52) |
| Severity of infection timepoint by perinatal timepoint | 0.82 (95% CI 0.70 to0.95). | 0.80 (0.69 to 0.94) |
| Mild COVID and anxiety at subsequent timepoint | -0.72, 95% CI -1.38 to -0.07* | -85 (-1.52 to -0.19)* |
| Moderate anxiety at subsequent timepoint | -0.55, 95% CI -1.29 to 0.20 | -0.74 (-1.51 to 0.29) |
| Severe anxiety at subsequent timepoint | 0.65, 95% CI -1.22 to 2.51 | 0.71 ( -1.18 to 2.60) |
| Likelihood participant was infected and anxiety at subsequent timepoint | 0.76, 95% CI 0.01 to 1.52 | 0.83 (0.09 to 1.57) |
| Very likely infected and anxiety at subsequent timepoint | 1.13, 95% CI 0.24 to 2.02 | 1.04 (0.17 to 1.92) |
| Severely ill and anxiety at subsequent timepoint | 2.23, 95% CI 0.86 to 3.60 | 2.22 (0.83 to 3.60) |
| Impact of COVID on mental health and perinatal timepoint | OR 0.90, 95% CI 0.84 to 0.94 | 0.88 (0.83 0.93) |
| Meet friends and family and anxiety | 1.02, 95% CI 0.21 to 1.83 | 0.91 (0.04 to 1.78) |
| Not avoiding public transport and COVID at subsequent timepoint (not at all vs completely) | (aOR 5.15, 95% CI 1.62 to 16.34) | 5.01 (1.60 to 15.65) |
| Not working from home and COVID at subsequent timepoint (not at all vs completely) | (aOR 3.24, 95% CI 1.69 to 6.21) | 2.73 (1.41 to 5.25) |
| No using phone to keep in touch and COVID at subsequent timepoint (mostly vs completely) | (aOR 2.58, 95 %CI 1.75 to 3.81) | 2.54 (1.71 to 3.78) |
| Not avoiding social gatherings and COVID at subsequent timepoint (mostly vs completely) | (aOR 1.86, 95% CI 1.25 to 2.76) | 1.71 (1.14 to 2.55) |
| Not avoiding public gatherings and COVID at subsequent timepoint (mostly vs completely) | (aOR 1.80, 95% CI 1.24 to 2.62) | 1.61 (1.10 to 2.34) |

*p<0.05

Tables S2a and S2b show the impact of the severity of COVID on anxiety at the same timepoint without (Table S2a) and with adjustment for the recruitment period (Table S2b).

**Table S2a.** **Severity of COVID and anxiety at the same timepoint**

|  |  | Anxiety symptoms | | |
| --- | --- | --- | --- | --- |
|  | N | Mean (SD) | Coefficient | Adjusted coefficient |
| No COVID | 3143 | 9.23 (7.29) | Ref | ref |
| Mild COVID | 671 | 8.32 (6.98) | -0.66 (-1.19 to -0.13)* | -0.58 (-1.10 to -0.05)* |
| Moderate COVID | 433 | 9.68 (7.52) | -0.08 (-0.71 to 0.55) | -0.44 (-1.06 0.18) |
| Severe COVID | 37 | 10.71 (8.45) | 0.07 (-1.50 to 1.64) | -0.31 (-1.84 to 1.23) |

^a^ Adjusted for age, marital status, ethnic group, general health and previous mental health problems. *p<0.05

**Table S2b. Severity of COVID and anxiety at the same timepoint with adjustment for recruitment period**

|  |  | Anxiety symptoms | | |
| --- | --- | --- | --- | --- |
|  |  |  | Coefficient | Adjusted coefficient |
| No COVID |  |  | Ref | ref |
| Mild COVID |  |  | -0.65 (-1.20 to -0.10)* | -0.62 (-1.16 to -0.08)* |
| Moderate COVID |  |  | -0.12 (-0.77 to 0.53) | -0.54 (-1.19 0.11) |
| Severe COVID |  |  | 0.04 (-1.56 to 1.64) | -0.32 (-1.89 to 1.25) |

^a^ Adjusted for age, marital status, ethnic group, general health and previous mental health problems. *p<0.05

Table S3a and S3b show the multivariate model of COVID exposure, perceived risk, impact on mental health and anxiety symptoms without (Table S3a) and with adjustment for period of recruitment (Table S3b).

**Table S3a. Multivariate model of COVID exposure, perceived risk, impact on mental health and anxiety symptoms**

| **Anxiety**  **(SAAS total)** | **Coef.** | **Standard**  **Error** | **t-value** | **p-value** | **95% Conf. Interval** | | **Sig** |
| --- | --- | --- | --- | --- | --- | --- | --- |
| **Ethnicity** |  |  |  |  |  |  |  |
| White | 0 | - | - | - | - | - |  |
| Asian/British Asian | 1.497 | .453 | 3.31 | .001 | .609 | 2.384 | *** |
| Black/African/Carribean | .598 | .786 | 0.76 | .447 | -.943 | 2.138 |  |
| Mixed/multiple ethnicity | 1.901 | .688 | 2.76 | .006 | .553 | 3.249 | *** |
| Other | .295 | 1.587 | 0.19 | .853 | -2.815 | 3.404 |  |
| **Previous mental health probs** | 3.6 | .282 | 12.75 | 0 | 3.047 | 4.153 | *** |
| **General health** | -.1 | .006 | -16.67 | 0 | -.112 | -.088 | *** |
| **COVID exposure** | | | | | | | |
| No COVID | 0 | . | . | . | . | . |  |
| Mild COVID | -.243 | .266 | -0.91 | .362 | -.764 | .279 |  |
| Moderate COVID | -.389 | .312 | -1.24 | .213 | -1.001 | .224 |  |
| Severe COVID | -.723 | .771 | -0.94 | .348 | -2.233 | .787 |  |
| **Perceived risk of them, their baby or someone close to them getting COVID** | | | | | | | |
| Unlikely | -.408 | .425 | -0.96 | .337 | -1.241 | .425 |  |
| Uncertain | -.345 | .413 | -0.83 | .404 | -1.154 | .465 |  |
| Likely | -.71 | .428 | -1.66 | .097 | -1.549 | .129 |  |
| Very likely | -.593 | .465 | -1.28 | .202 | -1.505 | .318 |  |
| **Perceived risk of them, their baby or someone close to them being severely ill with COVID** | | | | | | | |
| Unlikely | .194 | .324 | 0.60 | .548 | -.441 | .829 |  |
| Uncertain | .647 | .34 | 1.90 | .057 | -.019 | 1.314 |  |
| Likely | 1.572 | .441 | 3.57 | 0 | .708 | 2.437 | *** |
| Very likely | 1.235 | .65 | 1.90 | .058 | -.04 | 2.509 |  |
| **Impact of COVID on their mental health** | | | | | | | |
| Slight impact | 1.343 | .259 | 5.20 | 0 | .836 | 1.85 | *** |
| Moderate impact | 3.576 | .319 | 11.21 | 0 | 2.951 | 4.201 | *** |
| Severe impact | 5.68 | .475 | 11.96 | 0 | 4.75 | 6.611 | *** |

*** p<.01, ** p<.05. Adjusted for relationship status, ethnicity, age, general health and previous mental health problems. Information for adjusted variables that were significant is included in the table.

**Table S3b. Multivariate model of COVID exposure, perceived risk, impact on mental health and anxiety symptoms: Adjusted for time point and recruitment period**

| **Anxiety**  **(SAAS total)** | **Coef.** | | **Standard Error** | **t-value** | | **p-value** | **95% Confidence Interval** | | | | **Sig** |
| --- | --- | --- | --- | --- | --- | --- | --- | --- | --- | --- | --- |
| Time point early | ref | | . | . | | . | . | | . | |  |
| Mid | -1.522 | | .194 | -7.84 | | 0 | -1.902 | | -1.142 | | *** |
| Late | -2.192 | | .209 | -10.50 | | 0 | -2.601 | | -1.783 | | *** |
| Post partum | -1.733 | | .234 | -7.39 | | 0 | -2.192 | | -1.273 | | *** |
| Recruitment period | ref | | . | . | | . | . | | . | |  |
| 2 | .754 | | .49 | 1.54 | | .124 | -.207 | | 1.714 | |  |
| 3 | .623 | | .468 | 1.33 | | .183 | -.294 | | 1.54 | |  |
| 4 | .281 | | .529 | 0.53 | | .595 | -.756 | | 1.319 | |  |
| White ethnic grp | ref | | . | . | | . | . | | . | |  |
| Mixed/multiple eth~s | 1.636 | | .699 | 2.34 | | .019 | .266 | | 3.006 | | ** |
| Asian/Asian British | 1.486 | | .455 | 3.27 | | .001 | .595 | | 2.377 | | *** |
| Black/African/Caribean | .7 | | .798 | 0.88 | | .38 | -.863 | | 2.263 | |  |
| Other ethnic group | -.533 | | 1.628 | -0.33 | | .743 | -3.724 | | 2.657 | |  |
| Age | -.098 | | .028 | -3.44 | | .001 | -.154 | | -.042 | | *** |
| Previous mental health problems | Ref | | . | . | | . | . | | . | |  |
| 1 | 3.409 | | .291 | 11.71 | | 0 | 2.838 | | 3.979 | | *** |
| No covid | Ref | | . | . | | . | . | | . | |  |
| Mild | .007 | | .273 | 0.03 | | .979 | -.528 | | .542 | |  |
| Moderate | -.156 | | .322 | -0.48 | | .628 | -.788 | | .476 | |  |
| Severe | -.901 | | .777 | -1.16 | | .246 | -2.424 | | .621 | |  |
| General health | -.101 | | .006 | -16.77 | | 0 | -.113 | | -.089 | | *** |
| How likely you infection | ref | | . | . | | . | . | | . | |  |
| Unlikely | -.441 | | .431 | -1.02 | | .306 | -1.285 | | .403 | |  |
| Uncertain | -.205 | | .419 | -0.49 | | .625 | -1.026 | | .616 | |  |
| Likely | -.23 | | .435 | -0.53 | | .598 | -1.083 | | .624 | |  |
| Very likely | .137 | | .477 | 0.29 | | .774 | -.799 | | 1.073 | |  |
| How likely you severely ill | ref | | . | . | | . | . | | . | |  |
| Unlikely | .033 | | .325 | 0.10 | | .92 | -.604 | | .67 | |  |
| Uncertain | .486 | | .343 | 1.42 | | .156 | -.186 | | 1.157 | |  |
| Likely | 1.223 | | .444 | 2.75 | | .006 | .352 | | 2.094 | | *** |
| Very likely | 1.102 | | .67 | 1.64 | | .1 | -.212 | | 2.415 | |  |
| How much has COVID impact mh | ref | | . | . | | . | . | | . | |  |
| Slight impact | 1.479 | | .264 | 5.60 | | 0 | .961 | | 1.997 | | *** |
| Moderate impact | 3.451 | | .326 | 10.59 | | 0 | 2.813 | | 4.09 | | *** |
| Severe impact | 5.726 | | .48 | 11.93 | | 0 | 4.785 | | 6.667 | | *** |
| Constant | 17.2 | | 1.2 | 14.33 | | 0 | 14.847 | | 19.552 | | *** |
| Constant | 17.174 | | 1.025 | .b | | .b | 15.278 | | 19.306 | |  |
| Constant | 17.467 | | .541 | .b | | .b | 16.438 | | 18.559 | |  |
|  | | | | | | | | | | | |
| Mean dependent variable | | 8.996 | | | SD dependent var | | | 7.168 | |  |  |
| Number of observations | | 3562 | | | Chi-square | | | 1136.670 | |  |  |
| Prob > chi2 | | 0.000 | | | Akaike crit. (AIC) | | | 22030.419 | |  |  |
| **** p<.01, ** p<.05, * p<.1* | | | | | | | | | | | |
